# The Importance of Pathogen Load

**DOI:** 10.1371/journal.ppat.1004563

**Published:** 2015-01-08

**Authors:** Aubrey J. Cunnington

**Affiliations:** Section of Paediatrics, Medical School Building, St Mary's Campus, Imperial College, Norfolk Place, London, United Kingdom; The Fox Chase Cancer Center, United States of America

It seems obvious that the number of pathogens should be important in the pathogenesis of an infectious disease [1], [2]. The relationship between the pathogen load and severity is one of the most fundamental questions, and yet, strangely, one of the most difficult to answer [3]. One reason for this is that it is often rather difficult to determine the total pathogen load in an infected host, particularly in an infected human. Pathogens can be distributed, non-uniformly, throughout multiple different cell, tissue, or organ compartments of the body, many of which are difficult to sample. For this reason, we are often constrained by measuring pathogen load in the samples that are readily accessible such as blood, urine, and sputum, and we must assume that these are representative of total pathogen load. The success of this approach is borne out by the usefulness of these measurements to guide clinical management of patients with some infections [4], [5], but it is also well recognised that these methods are imperfect [6], [7]. Variations in both pathogen and host can alter distribution of the pathogen and the likelihood that a given pathogen burden will cause disease [1], [2], [8]–[11]. Imperfect estimation of pathogen load becomes a particularly important problem when trying to understand host responses to infection, and their role in pathogenesis. In order to interpret whether a host response is insufficient, appropriate, or excessive, it needs to be assessed in relation to the pathogen load that triggered it.

In this issue of *PLOS Pathogens*, Nicholas Anstey and colleagues present an analysis of host responses in relation to pathogen load in both *Plasmodium vivax* and *P. falciparum* malaria [12]. These authors have previously been instrumental in demonstrating that along with *P. falciparum*, *P. vivax* is an important cause of severe malaria, morbidity, and mortality [13], [14]. Their current findings help us to understand why and illustrate the importance of trying to determine pathogen load and distribution. Measurement of pathogen load in malaria might seem simple because illness only occurs during the phase of asexual parasite replication within red blood cells. Thus, malaria is often diagnosed by microscopic examination of a blood sample, and the simplest metric of pathogen load is assessment of parasitemia—the percentage of infected red blood cells (iRBCs). However, anemia is a common consequence of malaria, and so the pathogen burden in blood is more accurately quantified by calculation of the absolute parasite density in blood, taking account of the number of red blood cells per µL. Even this refinement can be very misleading with certain species of *Plasmodium* (notably *P. falciparum* and the common “mouse model” parasite *P. berghei ANKA*) because iRBCs can adhere to the endothelium, which lines small blood vessels (sequestration), resulting in their under-representation in circulating blood [15]. Methods have been developed to estimate total parasite load (parasite biomass) in *P. falciparum* malaria by measuring the plasma concentration of parasite molecules, which are released into the host circulation, and demonstrate that this is a much better predictor of severity than measurement of the circulating parasites alone [15]–[18]. In addition, the proportion of parasites that are sequestered is particularly high in some manifestations of severe malaria [11], [15], [16], [19]. Anstey and colleagues propose a method to approximate the total parasite biomass in *P. vivax* malaria using the plasma concentration of parasite lactate dehydrogenase (LDH) [12]. Similar to the established finding in *P. falciparum* malaria, total *P. vivax* parasite biomass appears higher in severe than uncomplicated malaria, and also appears to be underestimated by counting parasites in the peripheral blood. Since *P. vivax* iRBCs exhibit much less endothelial adhesion than *P. falciparum* iRBCs [20], the authors propose that *P. vivax* may accumulate outside of the endothelium-lined compartments of the blood, possibly in the slow open circulation of the spleen. This explanation is appealing, but there are some important caveats. First, *P. vivax* biomass is assumed to be approximately proportional to the plasma concentration of parasite LDH, but this will only be true if this molecule is released evenly throughout the parasite lifecycle or if there is an even distribution of life cycle stages at any point in time (i.e., the infection is totally asynchronous). Whilst all subjects had over-representation of younger parasite stages in the blood, there was no difference in this distribution between subjects with severe and uncomplicated *P. vivax* malaria, meaning that comparison of parasite LDH between groups should be valid. Second, the rate of production and clearance of parasite LDH could vary between subjects with severe and uncomplicated malaria, resulting in differences in plasma concentration that are not solely due to parasite biomass. Thus, parasite LDH probably only gives a semi-quantitative estimate of *P. vivax* biomass.

Despite these limitations, Anstey and colleagues are to be congratulated for applying this methodology to produce a unique assessment of the relationship between parasite biomass and the major determinants of severe malaria pathogenesis: inflammation, sequestration, and vascular endothelial dysfunction [15], [19]. Their data are all the more remarkable because they compare large numbers of healthy controls and subjects with both *P. falciparum* and *P. vivax* malaria [12]. Whilst there appear to be many similarities between severe disease caused by both parasite species, it is only when parasite biomass and distribution are considered that distinct pathogenic mechanisms begin to be revealed. In *P. vivax* malaria, the parasite LDH concentration correlates with the systemic inflammatory response, but not with markers of endothelial activation. In contrast, circulating parasitemia correlates much better with endothelial activation. This is intriguing because it suggests that somehow the circulating parasites are activating the vascular endothelium independent of soluble circulating factors (which would also arise from the non-circulating parasites). Does this mean that some sort of non-adhesive interaction between circulating iRBCs and endothelial cells triggers endothelial activation? Further research will be necessary to answer this question. But it may also provide insights into a longstanding debate about whether endothelial sequestration of *P. falciparum* is the cause, or a consequence, of endothelial activation [15], [21]. In humans with *P. falciparum*, these two phenomena are often so closely correlated that they are impossible to separate, but we now know that for *P. vivax* at least, endothelial activation can occur independent of endothelial parasite sequestration.

Whilst the study by Anstey and colleagues clearly illustrates the importance of assessing pathogen load [12], even this approach is oversimplified. Quite apart from the complexities of how and where to measure pathogen load, there is also the question of when to measure it? In naturally acquired, serious infections in humans, we rarely have a choice in this matter—we can only measure it at the time they present for treatment. But the pathogen load at this point in time is the consequence of a dynamic interplay between the rate of pathogen replication, how long the pathogen has had to replicate (the number of replication cycles) before the person seeks medical attention, and how effectively the host response constrains pathogen replication. Beyond measuring pathogen load, in the future we need to consider new methods that will allow us to interpret pathogen load in this dynamic context.
